# Creutzfeldt-Jakob disease mimicking autoimmune encephalitis with CASPR2 antibodies

**DOI:** 10.1186/s12883-014-0227-7

**Published:** 2014-11-30

**Authors:** Frédéric Zuhorn, Almut Hübenthal, Andreas Rogalewski, Müjgan Dogan Onugoren, Markus Glatzel, Christian G Bien, Wolf-Rüdiger Schäbitz

**Affiliations:** Department of Neurology, Evangelisches Krankenhaus, Burgsteig 13, Bielefeld, 33617 Germany; Epilepsy Center Bethel, Krankenhaus Mara, Maraweg 17-21, Bielefeld, 33617 Germany; Institute of Neuropathology, Universitätsklinikum Hamburg-Eppendorf, Martinistraße 52, Hamburg, 20246 Germany

**Keywords:** CASPR2, VGKC, Creutzfeldt-Jakob disease, Encephalitis, Autoimmune, Autoantibody, Thyroperoxidase antibodies

## Abstract

**Background:**

Differential diagnosis of severe progressive dementia includes a wide spectrum of inflammatory and neurodegenerative diseases. Particularly challenging is the differentiation of potentially treatable autoimmune encephalitis and Creutzfeldt-Jakob disease. Such a coincidence may indeed complicate the correct diagnosis and influence subsequent treatment.

**Case presentation:**

A 75-year-old woman was admitted due to rapid progressive cognitive impairment. Her husband observed a temporal disorientation and confusion. The initial neurological examination and an extensive neuropsychological evaluation showed significant impairments in almost all tested cognitive domains. All other neurological functions including motor, sensory and coordinative function were intact. Initial diagnostics included EEG, MRI and lumbar puncture with unspecific results. Complementary blood testing revealed a positive result for antineural antibodies to Contactin-associated protein 2 (CASPR2) and the patient received treatment for CASPR2 autoimmune encephalitis. Further symptoms and results, including 14-3-3 proteins, led to suspected Creutzfeldt-Jakob disease. The postmortem examination supported the diagnosis of a definitive Creutzfeldt-Jakob disease.

**Conclusion:**

One could argue that global screening for antineural antibodies may lead to a false diagnosis triggering intense and potentially dangerous procedures. We believe, however, that potentially treatable causes of dementia should aggressively sought out and subsequently treated in an attempt to curtail the course of disease and ultimately reduce the rate of mortality.

## Background

Differential diagnosis of severe progressive dementia includes a wide spectrum of inflammatory and neurodegenerative diseases. Particularly challenging is the differentiation of potentially treatable autoimmune encephalitis and Creutzfeldt-Jakob disease (CJD). Such a coincidence may indeed complicate the correct diagnosis and influence subsequent treatment. The diseases can mimic one another to such an extent that one is led to believe that the patient is suffering from CJD when he or she has in fact a treatable autoimmune encephalitis [1-3] or vice versa [2,4,5]. None of these VGKC complex antibodies in CJD patients have been found to be directed to LG1 or CASPR2, yet.

We here report a recent case of **C**ontactin-**as**sociated **pr**otein 2-Antibody (CASPR2) production during ongoing CJD, discuss the potential biological causes and the clinical consequences.

## Case presentation

**Figure 1:**
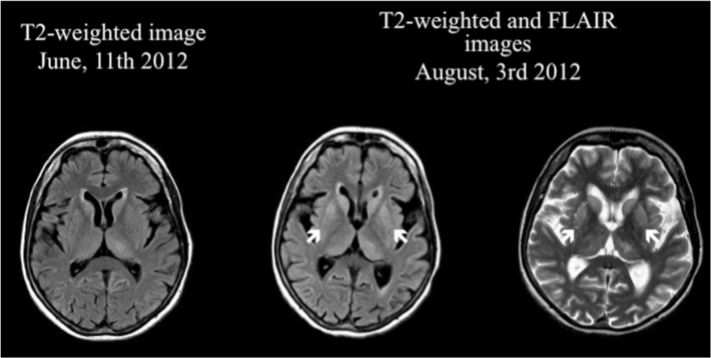
**MRI formation of symmetrical hyperintensities in the putamen and caudate head within two months seen on T2-weighted and FLAIR images.**

**Figure 2:**
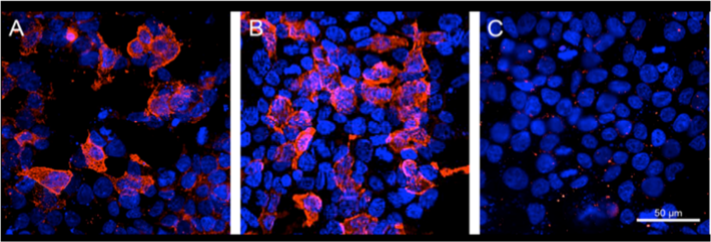
**Cell based assays for demonstration of CASPR2 antibodies (Euroimmun, Lübeck, Germany). (A)** Serum of patient diluted 1:15 incubated with HEK cells transfected with CASPR2; the antibodies are visualized by a Alexa 594 anti-human-IgG antibody; mild counterstaining with Hoechst 33342. **(B)** Serum of a patient with classical limbic encephalitis and CASPR2 antibodies, technical details as in A. **(C)** Serum of patient incubated with control cells not expressing CASPR2 (negative result), technical details as in A. These images demonstrate that the patient’s serum does not bind non-specifically to the CASPR2 expressing HEK cells in A.

**Figure 3:**
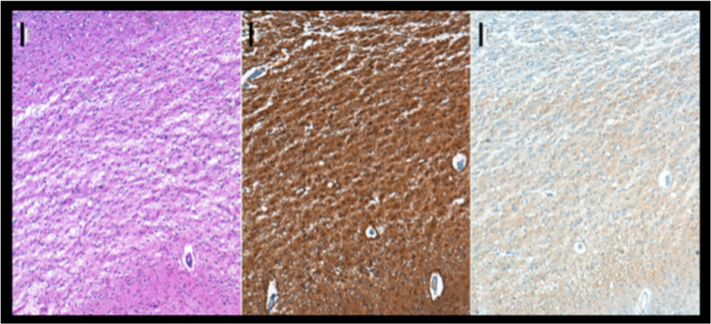
**Frontal cortex (lower side towards pia mater; upper side towards white matter), PrP; Scale bar 100 um.** Note the confluent spongiosis and the diffuse synaptic PrP deposits. Brains were fixed in 4% formalin and paraffin-embedded tissue samples of frontal cortex were cut into 3 μm thick serial sections, mounted on glass slides and processed for immunhistochemical staining using specific antibodies to PrP. Visualization of primary antibody was achieved using the diaminobenzidine streptavidin-biotin horseradish peroxidase method on an automated stainer (Ventana/Roche).

A 75-year-old woman was admitted to our hospital due to rapid progressive cognitive impairment. During the previous year, the patient had shown mild cognitive impairment due to moderate leukoariosis, thought to be associated with arterial hypertension and hypercholesterolemia. There was no positive family history of dementia or dementia-like symptoms in the anamnesis. Ten days before admission, her husband observed a temporal disorientation and confusion, e.g. the patient could not recall the present date and put a saltshaker into the refrigerator. When admitted to our care the patient was conscious but disorientated to place, time and person. The initial neurological examination and an extensive neuropsychological evaluation showed significant impairments in almost all tested cognitive domains including attention, concentration, memory, executive function and visual-constructional ability. In addition, there was evidence of a right-sided visual neglect, aphasia in terms of language comprehension disorder and pronounced apraxic impairments corresponding to dysfunctions of her left-sided parietal circuits. All other neurological functions including motor, sensory and coordinative function were intact. An initial electroencephalography showed unspecific encephalopathy patterns. The MRI showed multiple microangiopathic lesions: left-sided lesions in the thalamus, parietooccipital, temporo mesial, thalamic, frontal and parietal cortices, as well as right-sided lesions in the basal ganglia. The brain-SPECT showed hypometabolism in the frontoparietal and parietooccipital cortices, more obvious on the left side, with normal nuclide accumulation in motor and occipital cortex. The primary investigation of cerebrospinal fluid revealed a pleocytosis of 7 Leukocytes/μl [<5 Leukocytes/μl] with a Total Protein of 701 mg/l [<450 mg/l] and 2,31 mmol/l Lactate [1,2-2,1 mmol/l]. Simultaneously, thyroperoxidase antibodies (serum titre 1606 IU/ml [<60 IU/ml]) were detected. It was initially concluded based on these results that the patient was suffering from autoimmune encephalitis, believed to be caused by autoimmune thyroiditis. High dosage intravenous methylprednisolone therapy was initiated [6]. Despite treatment however, the patient continued to exhibit cognitive and neuropsychological symptoms and presented the first tonic-clonic seizure, leading to the initiation of Levetiracetam therapy. The ongoing diagnostic workup included a broad search for potential autoimmune diseases. Serum and CSF were tested for antibodies to the following antigens: CASPR2, LGI1, NMDAR, GAD65, GAD67, GABABR, AMPAR1/2, GlyR and onconeural antigens, whereby CASPR2-antibodies were detected (serum titre 1:2000, see Figure 1A; no antibody studies in CSF done). This result was interpreted as support for the hypothesis of ongoing autoimmune encephalitis. Treatment was now escalated to eight tryptophan immunoadsorptions processing two liters plasma per session. Although immunoadsorption effectively reduced the titre of CASPR2-antibodies (serum titre 1:32), the patient’s cognitive and general neurological condition worsened. A positron emission tomography was now added to disclose malignancies. Apart from cystic structures in kidneys and liver, no underlying oncological disease was detected. Four weeks after the first MRI, follow up imaging now revealed new hyperintensities in the basal ganglia and both dorsal thalami [Figure 2 right]. Furthermore, the EEG now presented a generalized periodic pattern with triphasic waves. Continuous CSF studies now showed normalization of Leukocytes (1/μl) and Total Protein (292 mg/l), but increased Tau and 14-3-3 proteins leading to the suspicion of a possible Creutzfeldt-Jakob disease [7,8]. The patient continued to detoriate over the following month after discharge, dying approximately one year after the onset of symptoms. The postmortem examination showed signs of spongiform encephalopathy [Figure 3], supporting our diagnosis of a definitive Creutzfeldt-Jakob disease [7,8].

### Discussion

A rapidly progressive dementia with symptoms such as disorientation, apraxia, aphasia, extrapyramidal dysfunction, and psychiatric symptoms caused by CJD may mimic treatable types of autoimmune encephalitis. As shown in the present case, thyroperoxidase- and CASPR2-antibodies known to be associated with cognitive and neuropsychological symptoms [9], can accompany the devastating spongiform encephalopathy.

Our findings expand upon prior reports in which VGKC complex antibodies were shown to be associated with CJD. In previous studies, the patient had not been tested for CASPR2 or LG1 [4] and the actual antigen could not be identified. In addition, a recently published study [2] demonstrated neuronal surface antigens in patients’ CSF with rapid neurological deterioration. Patients in this study suffered from different non-specific cognitive deficits with variable degrees of memory loss and confusion. Interestingly, none of the patients in this series with definite CJD displayed antibodies against neuronal surface antigens. Those few patients (1,7%) in whom neuronal surface antigens were detected did not fulfill the diagnostic criteria for probable or possible CJD. This is in contrast to our finding, in which CASPR2-antibodies [Figure 1] in serum were clearly associated with a positive test for 14-3-3 protein and later on confirmed definite sporadic CJD by postmortem neuropathological analysis. It should be noted that the first positive finding of CASPR2-anibodies has been validated by a second test for CASPR2-antibodies, in which the serum titre, parallel to the decreasing CSF cell count, was significantly lower as a result of immunoadsorption treatment.

Another case series reported about a 68-year-old patient with sporadic CJD tested positive for serum antibodies to VGKC and GlyR antibodies but negative for CASPR2 and LG1 antibodies [5].

## Conclusion

The biological cause of this molecular mimicry is currently unknown and deserves further investigation. One may hypothesize that prion-induced neurodegeneration may have triggered an immunological reaction in peripheral lymph nodes causing antibody production against certain neuronal structures such as elements of voltage gated potassium channels.

With the diagnosis still unconfirmed, we chose to initiate therapy for the potentially treatable differential diagnosis. One could argue that global screening for antineural antibodies led us to a false diagnosis triggering intense and potentially dangerous procedures. We believe, however, that potentially treatable causes of dementia should be aggressively sought out and subsequently treated to avoid poor outcome. In fact, encephalitic syndromes are treatable conditions that may mimic CJD [1,3].

The development of a CJD case into a progressive and untreatable encephalopathy, as presented here, is certainly very rare. Nevertheless, such unique cases are important as they present new opportunities to study the complex role of the immune system in the CNS.

### Consent

Written informed consent was obtained from the legal guardian of the patient for publication of this Case report and any accompanying images. A copy of the written consent is available for review by the Editor of this journal.

## Abbreviations

CASPR2: Contactin-associated protein 2
CSF: Cerebrospinal fluid
CNS: Central nervous system
CJD: Creutzfeldt-Jakob disease
EEG: Electroencephalography
FLAIR: Fluid attenuated inversion recovery
GlyR: Glycine receptor
HEK: Cells human embryonic kidney cells
LG1: Leucine-rich, glioma-inactivated 1
MRI: Magnetic resonance imaging
PrP: Prion protein
SPECT: Single-photon emission computed tomography
VGKC: Voltage-gated potassium channel complex
